# Real-Time Monitoring and Control of Nanoparticle Formation

**DOI:** 10.1021/jacs.3c02484

**Published:** 2023-07-17

**Authors:** Yujie Guo, Vivien Walter, Steven Vanuytsel, Christopher Parperis, Jason T. Sengel, Eve E. Weatherill, Mark I. Wallace

**Affiliations:** † Department of Chemistry, King’s College London, Britannia House, 7 Trinity Street, London SE1 1DB, U.K.; ‡ Department of Engineering, King’s College London, London WC2R 2LS, U.K.

## Abstract

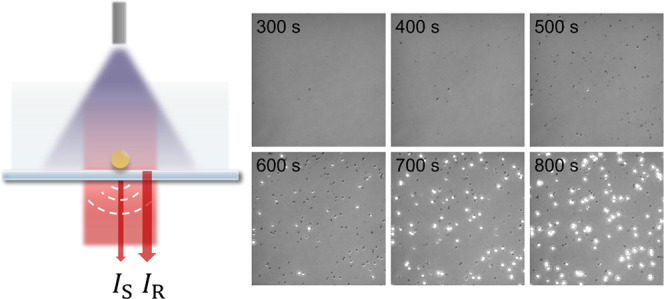

Methods capable of controlling synthesis at the level of an individual nanoparticle are a key step toward improved reproducibility and scalability in engineering complex nanomaterials. To address this, we combine the spatially patterned activation of the photoreductant sodium pyruvate with interferometric scattering microscopy to achieve fast, label-free monitoring and control of hundreds of gold nanoparticles in real time. Individual particle growth kinetics are well-described by a two-step nucleation–autocatalysis model but with a distribution of individual rate constants that change with reaction conditions.

## Introduction

The tunable material and optical properties of nanoparticles (NPs) have led to their rapid and widespread adoption, impacting areas as varied as catalysis,^1−3^ drug delivery,^4−7^ photothermal therapy,^8,9^ and biosensing.^10−13^ Unsurprisingly, nanoscopic dimensions are the key to their unique properties, and precise control of NP size and size dispersity is critical to their effective applications.^14−16^ Designing complex NPs with defined properties thus requires both a detailed understanding of NP growth and the means to control it.

Methods to monitor NP growth typically derive kinetics from ensemble properties that reflect nanoscopic changes in particle size: for example, absorption spectroscopy^17^ and dynamic light scattering^18^ are commonly used to give information about the size, dispersity, and state of aggregation. However, this information can only reflect the properties of the ensemble average, and the essential details of the particle-to-particle heterogeneity are lost.^19^

Examining how individual NP properties are distributed is a powerful route to better understand how to engineer such properties. For example, single-particle inductively coupled plasma mass spectrometry is an established method to sample the changes in NP mass distribution.^20^ The distribution of NP dimensions themselves can also be measured directly using techniques such as scanning electron microscopy and transmission electron microscopy, but despite its exquisite spatial resolution, electron microscopy typically can only provide “snapshots” of NPs during growth that do not permit the evolution of individual particles to be followed. Additionally, NPs are usually separated from the solution and dried before imaging,^21^ introducing further problems of continued growth and agglomeration during sample preparation.^22,23^

Several methods have sought to span this divide between well-characterized (but ensemble) kinetics and nanoscopically resolved (but static) single-particle imaging. In particular, the same optical properties that provide ensemble-averaged readout of particle growth can also be applied to individual NPs.^24^ For example, the change in surface plasmon resonance with particle length can be used to monitor gold nanorod oxidation^25^ and growth.^26^ Although powerful, such methods rely on the presence of length-dependent plasmon resonance. Photothermal microscopy provides an important alternative route to achieve NP detection, measuring the signal produced by the temperature-induced refractive index change. Conventional photothermal microscopy has integration times of the order of the millisecond per pixel, which limits its kinetic applications.^27,28^ Wide-field photothermal microscopy has improved the acquisition rate, but to date, at the expense of some sensitivity.^29−31^ Atomic force microscopy can also be used to resolve gold nanoparticle (AuNP) growth with subnanometer spatial resolution but is constrained by its relatively slow temporal resolution.^32^ Looking forward, recent advances in liquid-cell electron microscopy provide one promising route to improve on these methods, with reports of atomically resolved real-time imaging of the rotation^33^ and formation^34^ of individual NPs, but this is currently at the expense of complex, expensive instrumentation and low throughput.

Individually, these methods address many of the requirements for a ubiquitous and easily applied monitor of NP kinetics. However, none are able to fully satisfy the requirements of (1) a particle-by-particle measurement of growth with high throughput; (2) real-time reporting on NP size with high sensitivity and high spatiotemporal resolution; and (3) a readout independent of the specific optical properties of the NP.

**Figure 1:**
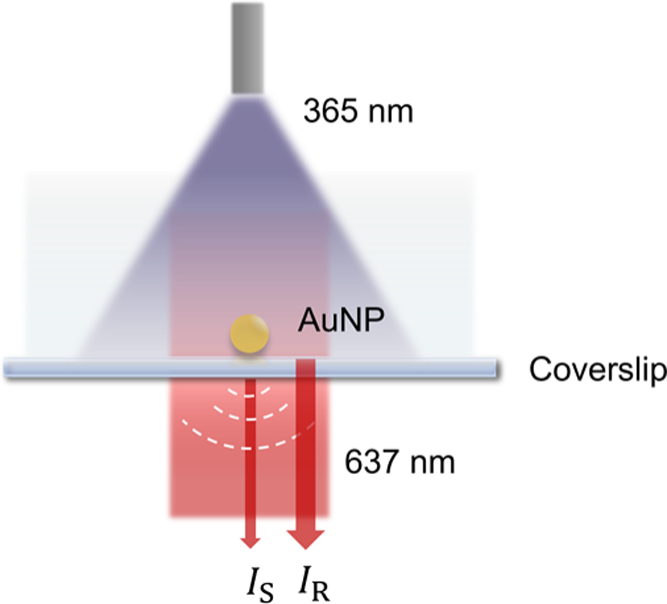
iSCAT detection of NP formation. Interference between light scattered from a growing NP (*I*_S_) and light reflected at the water–glass interface (*I*_R_) is detected. For experiments using sodium pyruvate as a photoreductant, a 365 nm LED light is delivered via a liquid light guide.

Here, we apply interferometric scattering microscopy (iSCAT) as a simple label-free method to monitor the growth kinetics of individual NPs in real time. As shown in Figure 1, the sensitivity of iSCAT is enabled by coupling the weak light scattering from an individual particle (*I*_S_) to an external reference field (*I*_R_) provided by the reflection from a surface, in this case, a glass coverslip. The sensitivity of iSCAT is illustrated by its ability to resolve individual biomolecules down to approximately 40 kDa in mass.^35,36^ iSCAT also often exploits NPs as labels to achieve high-speed tracking at the nanoscale;^37,38^ we reasoned that it would also be straightforward to use this method as a probe of NP formation itself.

We chose AuNP synthesis via chemical reduction as a well-understood archetype to validate our method.^39−41^ In the classical method of Turkevich, citrate acts as both reductant and templating agent,^42,43^ with kinetics generally well-described by two-step nucleation–autocatalysis.^44^ This simple two-step model, suggested by Finke and Watzky (hereafter the FW-model), offers a direct relation between particle size and kinetics (eq 1):
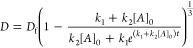
1where *D* is the diameter of an individual AuNP at the given time *t*, *D*_f_ is the final diameter, *k*_1_ and *k*_2_ are the rate constants of nucleation and autocatalytic growth, respectively, and [*A*]_0_ is the initial gold precursor concentration.

Since our aim is to tailor NP properties in situ, a means of direct control of NP formation is also required alongside single NP monitoring. To achieve this, we use spatially patterned photoinitiation to provide spatiotemporal control and ambient initiation of NP formation. Photoreduction is an established method for NP preparation,^45−48^ and here we apply the photoreductant control of AuNP formation using sodium pyruvate (SP); SP is an efficient photoreductant recently used in the polymer synthesis^49,50^ but to the best of our knowledge previously not used for NP formation. Together, monitoring via iSCAT and tight photocontrol via SP enable us to deliver real-time label-free monitoring and control of single NP growth and kinetics.

## Results and Discussion

### Monitoring Single AuNP Growth

AuNP growth is tracked using a custom iSCAT microscope (Figures 1 and S1). Briefly, a 637 nm fiber-coupled and homogenized multimode diode laser is transmitted through a 100 × microscope objective illuminating a chambered coverslip (Figure S1C). Light scattered from growing AuNPs on the coverslip surface interferes with light reflected from the coverslip interface and is imaged on a scientific CMOS camera. Laser power is adjusted to utilize the full well capacity of the detector for a desired frame rate. Images are then background-corrected and processed to calculate the temporal evolution of contrast for each particle. Further details of apparatus, methods, and processing are provided in the Supporting Methods.

**Figure 2:**
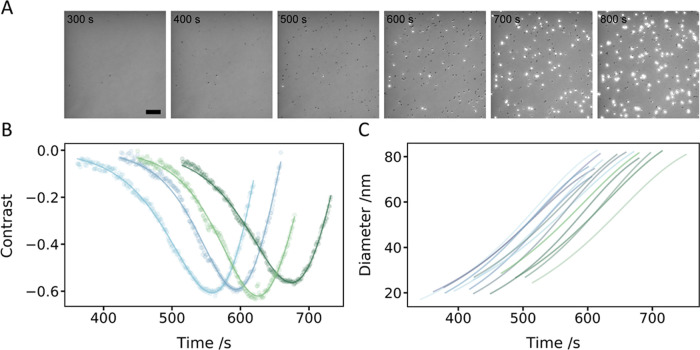
iSCAT monitoring of single AuNP growth. (A) Montage of images during AuNP growth using citrate as the reductant (scale bar 3 μm). (B) Contrast evolution of 4 particles fit to the FW-model (dots represent raw contrast reported by iSCAT; solid lines are fitted data). (C) Size evolution of 14 particles.

Figure 2 (and Movie S1) depicts a typical NP growth experiment: 0.4 mM of HAuCl_4_ was mixed with 1 mM of citrate solution on ice for 5 min and then added to the chambered coverslip and sealed with a second coverslip before imaging. Typically, kinetics from around 200 particles at a density of 0.5 particles per μm^2^ were recorded with a maximum temporal resolution of 100 μs.

Particle contrast, *c*, can be expressed in terms of the particle scattering cross section (σ_scat_), a proportionality constant (β) describing the instrument sensitivity and the Gouy phase shift between reference and scattered fields (φ):^51^
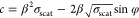
2σ_scat_ depends on particle size; thus, as NPs grow, they are first detected with contrast more negative than the overall background (dark spots) before becoming positive (bright spots) (Figure 2A,B).^52,53^

The evolution of contrast with time for four representative particles is shown in Figure 2B. These raw data are fitted (solid lines) by linking the diameter dependence in the FW-model (eq 1) to the expected diameter dependence of σ_scat_ (and thus contrast) for spherical particles (eq 2 and Supporting Methods). From fitting this dependence, the evolution of particle diameter with time can then also be plotted (Figure 2C). For the experiment shown in Figure 2, we chose imaging conditions to probe the early stages of growth; beyond 80 nm, the signal from individual NP becomes saturated on the detector. Overall, NP growth kinetics are well-described by the FW-model (Figure 2B).

### NP Kinetics

With our method established, we next examined the distribution of individual NP kinetics corresponding to the predictions of the FW-model.

**Figure 3:**
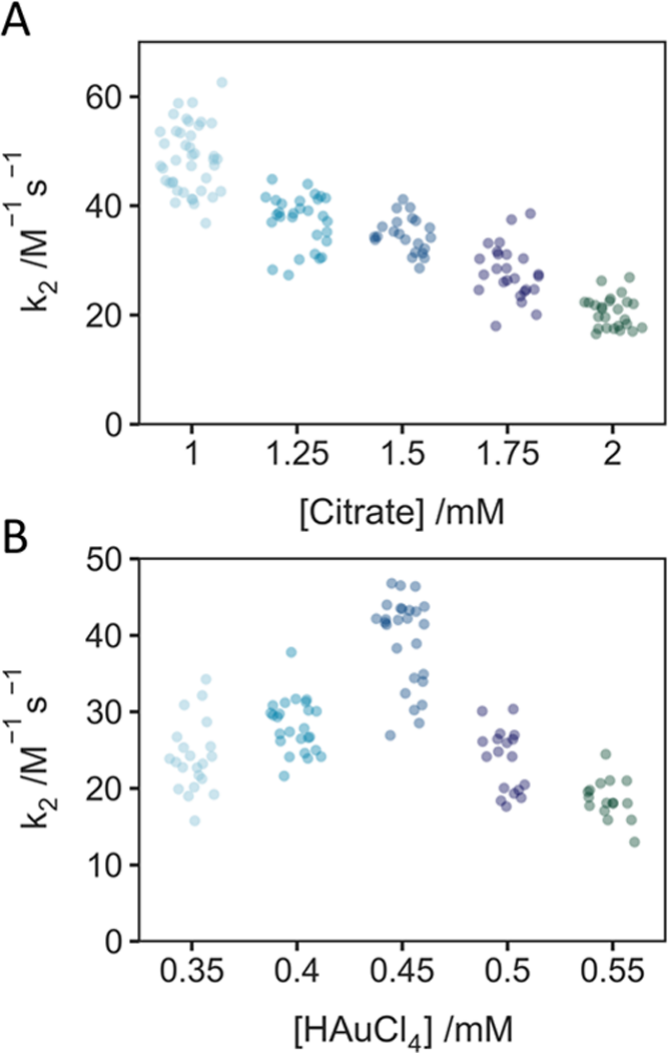
AuNP formation via citrate reduction. (A) Citrate dependence (0.4 mM HAuCl_4_). (B) HAuCl_4_ dependence (1.5 mM citrate).

To highlight our results, we present the rate constant distribution for autocatalytic growth (*k*_2_) and its dependence on sodium citrate and gold(III) chloride concentration (Figure 3). Each datum in Figure 3 reports *k*_2_ for a single AuNP. Figure 3A,B highlights the underlying heterogeneity in the population of the kinetics of NP growth. Given our initial conditions, marked variations in *k*_1_ and *D*_*f*_ are not expected; distributions for all fitting parameters are provided as the Supplementary Information (Figures S4 and S5).

We observe a decrease in the modal rate constant for autocatalysis (*k*_2_) as the citrate concentration is increased (Figure 3A). The decrease in the modal rate constant is perhaps at first glance unexpected: surely more reductant would result in faster autocatalytic growth? However, this result is consistent with citrate’s important role as a pH mediator in this reaction;^54^ it is a weak base, and thus as the pH of the reaction solution increases (increased citrate concentration), the reactivity of Au(III) complexes decreases and the formation rate of AuNP is expected to decrease.^54,55^

The variation of the autocatalysis rate constant with HAuCl_4_ concentration is more complex and thus more interesting (Figure 3B); 0.45 mM is a turning point before which the autocatalytic growth rate increases with the concentration of precursor. Then, as the concentration of HAuCl_4_ continues to increase, the concentration of citrate becomes the dominant factor in constraining the growth rate. As a result, *k*_2_ finally decreases with increasing precursor concentration.

### Establishing Photocontrol of NP Kinetics Using SP

Although the reductive formation of AuNPs by citrate is an obvious benchmark with which to test our experiment, its kinetics are complicated by the dual role of citrate as a reductant and templating agent in the reaction. Ensemble control of NP growth via changes in reductant concentration also relies on the repeatable timing and tight control of solution concentration. To achieve tight control over the reaction at the level of individual NPs, we exploited SP as a photoreductant.

**Figure 4:**
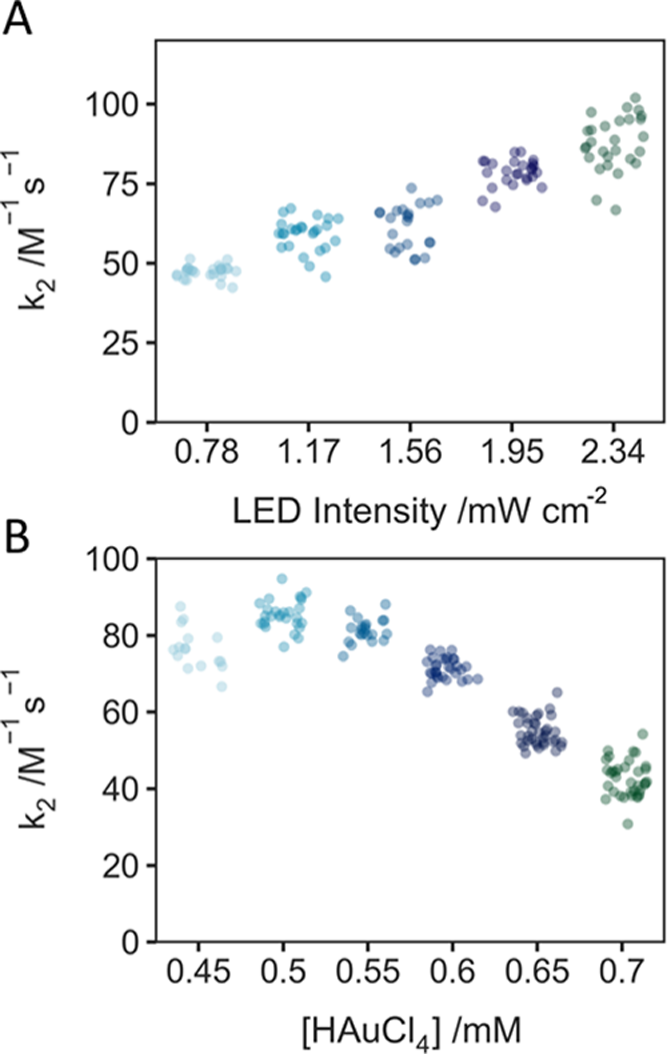
SP photocontrol of AuNP formation. (A) LED intensity dependence (0.6 mM HAuCl_4_, 0.35 mM SP). (B) HAuCl_4_ dependence (0.35 mM SP, LED intensity: 1.17 mW cm^–2^).

We repeated the experiments of Figure 3 using a 365 nm LED irradiation for photocontrol and replacing citrate with SP (0.35 mM SP; cf. 1–2 mM citrate). As expected (and in contrast to the case for citrate), Figure 4A shows a growth rate dependent on LED intensity and thus reductant generation. A similar trend in the HAuCl_4_ concentration dependence of *k*_2_ as with citrate was observed, with an initial increase in *k*_2_ followed by a decrease at higher HAuCl_4_ concentrations. The distributions for other fitting parameters are again provided in the Supporting Information (Figures S6 and S7).

SP photoreduction enables us to separate the roles of reductant and templating agent. Using 10 kDa polyethylene glycol (PEG10k), we also examined the effect of capping agent concentration in the presence of SP and observed a decrease in the NP diameter with the increase of capping agent concentration, as expected (Figure S8).

### Spatiotemporal Photocontrol of Single NP Growth

The use of SP also gives us control of where and when AuNP growth occurs. To exemplify this, the 365 nm LED illumination was replaced with a 405 nm laser epi-illumination patterned using a spatial light modulator (SLM, Figure S1A).

**Figure 5:**
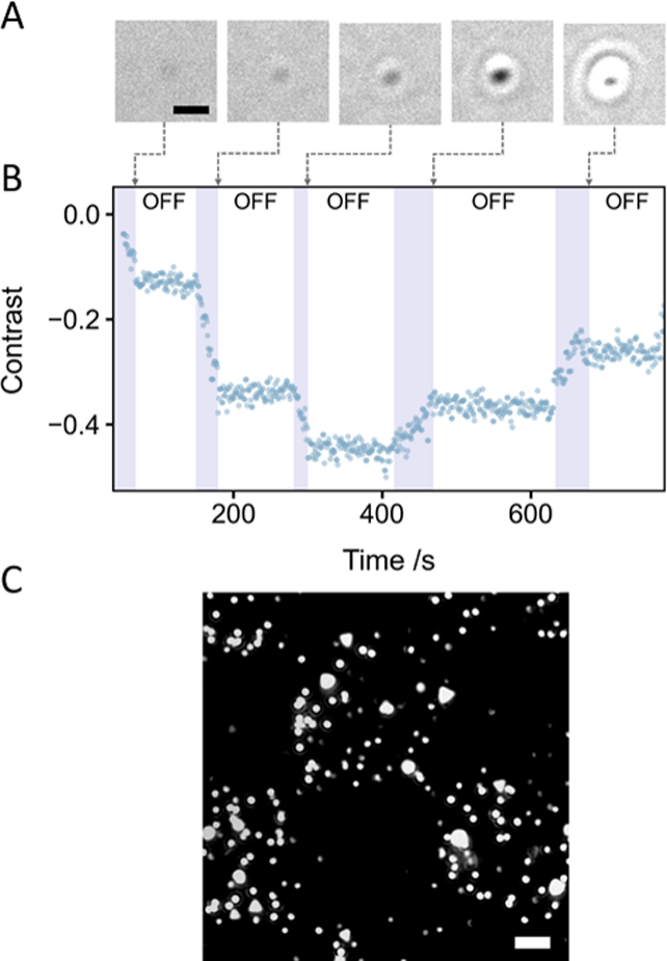
Spatiotemporal photocontrol of AuNP growth. (A) Montage of images during a single AuNP growth with a switched 405 nm illumination (scale bar 500 nm). (B) 405 nm illumination activates SP and enables particle growth. Exposure is indicated in purple. (C) A checkerboard mask was applied to the 405 nm illumination, using a SLM (scale bar 2 μm).

AuNP synthesis using SP was conducted under an alternating 405 nm laser irradiation. As shown in Figure 5A,B, the overall evolution of particle contrast was as expected (contrast first becoming more negative, before becoming more positive, as in Figure 2B). When the 405 nm laser was turned on, AuNP growth occurred, but growth was arrested in the absence of a 405 nm illumination.

Alongside temporal control, spatial control is also straightforward. Figure 5C shows the result of displaying a 8 × 8 μm checkerboard pattern of a 405 nm illumination on the coverslip surface, with AuNP growth only in the exposed areas.

## Conclusions

Our results establish the combination of label-free iSCAT microscopy and photoreductant spatiotemporal control as a simple means of studying NP kinetics in real time at the level of individual particles in a manner that is independent of the optical properties of the NP. Our measured values of *k*_2_ are similar to those previously reported in other measures of photochemical-initiated AuNP growth.^56,57^ Overall, our method is validated by testing the FW-model and retrieving the particle-by-particle distribution of kinetic parameters. Taking into account the differences between our experimental conditions and those previously published, these parameters are broadly comparable.^32,56,58^

This method is not without limitations. The advantages conveyed through label-free detection also mean that *all* scattering sources yield a detectable signal; for core–shell particles, for example, there could be little difference in particle contrast from different layers. Similarly, we require a reference beam generated by reflection from a surface; this essentially limits this method to studying NP growth at or near an interface.

In these experiments, our maximum NP density (approximately 0.5 μm^–2^) is essentially governed by the diffraction limit. Although iSCAT is capable of subnanometer precision measurements of object location,^59^ high densities are still challenging. Super-resolved variants of iSCAT microscopy are in their infancy,^60,61^ and to the best of our knowledge, they only offer spatial resolutions at length scales of approximately 100 nm, larger than might be useful for close-packed plasmonic NPs, for example.

On balance, despite these limitations, iSCAT brings significant advantages: (1) small (20 nm) AuNPs have previously been tracked using iSCAT down to a 2 μs temporal resolution,^38^ a time resolution essentially limited by detector technology; (2) AuNPs down to 2 nm in size are also routinely imaged using iSCAT.^53^ Although sensitivity is dependent on the particle polarizability, and thus its refractive index, iSCAT has also been applied to detect biomolecules with mass changes as small as 4 kDa.^62^ Our experiments have focused on collecting a large data set for AuNP growth over a relevant size scale. Overall, these data suggest that further study probing the kinetics at the very earliest stages of NP formation should also be attainable; (3) here, we have focused on gold as a well-established demonstrator; however, this method is applicable to any NP with a refractive index different from the surrounding medium.

Overall, these experiments suggest a route for the quantitative monitoring and control required for future routes to the precision engineering of individual NP properties. Tools such as these help better understand the properties of individual NPs, informing the design of more effective and targeted NPs for specific applications. The ability to design and synthesize new and more complex NPs is critical for advancing the field of nanotechnology and realizing the full potential of NPs for drug delivery, plasmonics, and smart materials.

## Experimental Section

### Materials

Gold(III) chloride trihydrate (HAuCl_4_·3H_2_O, ≥99.9%), sodium citrate tribasic dihydrate (Na_3_C_6_H_9_O_9_·2H_2_O), sodium pyruvate (SP, C_3_H_3_NaO_3_, ≥99%), poly(ethylene glycol) (PEG, averaged molecular weight: 10 000), and 60 nm citrate-capped AuNPs were purchased from Merck and used as received. 10, 20, 30, and 50 nm of PEG-carboxyl-capped monodispersed AuNPs were purchased from nanoComposix and used as received.

### Instrument Construction

A purpose-built iSCAT microscope comprising a 637 nm fiber-coupled and homogenized square-profile multimode diode laser (RLM-6000L, Kvant lasers, Slovakia) imaged the sample using a 100 × microscope objective (Plan-Apo 1.45NA, Nikon, Japan) (Figure S1), providing a 20 × 20 μm field of view. A chambered coverslip (Figure S1C) was illuminated and imaged onto a high-speed CMOS camera (PCO.dimax CS1, Excelitas PCO GmbH, Germany). Focus control was provided by a piezo-stage (P-545-3R8S, Physik Instrumente, Germany). The camera, laser, and piezo-stage were controlled using a custom LabVIEW program (National Instruments, USA).

LED illumination was delivered via a liquid light guide (pE4000/pE1906, CoolLED, UK) placed 10 mm above the reaction chamber. Spatial patterning was achieved via a 405 nm single mode diode laser illumination of a SLM (SN 4719, Meadowlark Optics, USA) combined into the iSCAT beam path via a dichroic filter (Di01-E405, Semrock, USA) (Figure S1).

### Contrast Calibration

Glass coverslips (24 mm × 60 mm, #1.5 thickness, Epredia) were washed successively for 15 min in chloroform, acetone, and isopropanol before drying with N_2_. Then, the solvent-cleaned coverslips were treated with oxygen-plasma for 6 min (Diener Electronic, Femto). Circular silicone spacers (ϕ9 × 2.5 mm thickness, Merck) were washed with the same protocol and dried under vacuum.

AuNP standards were sonicated for 1 min before a 25 μL sample solution was spin-coated onto the plasma-cleaned coverslip (4000 rpm, 30 s). A cleaned spacer was mounted onto the AuNP-coated coverslip, deionized water was added into the reaction chamber, and then sealed with an additional coverslip (22 mm × 22 mm, Merck), which was solvent-cleaned using the same procedure as introduced above. A laser power density of 0.003 mW μm^–2^ at 637 nm, a camera exposure time of 220 μs, and an overall time-lapsed frame rate of 3670 s^–1^ were selected.

### iSCAT Imaging of AuNP Growth

6 mM HAuCl_4_ and 30 mM citrate/SP stock solutions were freshly prepared and stored on ice. The final concentration of HAuCl_4_ and citrate/SP was varied by adjusting the volume of stock solutions added to each reaction to reach a final volume of 5 mL. During mixing, to minimize AuNP formation before imaging, the reaction solution was mixed in a light tight ice bath for 5 min. 150 μL of reaction solution was then sealed in the reaction chamber using the second coverslip placed on top of the spacer (Figure S1C). For citrate reduction, a laser power density of 0.003 mW μm^–2^ at 637 nm, a camera exposure time of 220 μs, and an overall time-lapsed frame rate 1 s^–1^ were selected. For SP reduction, a laser power density of 0.37 × 10^–5^ mW μm^–2^ at 637 nm, a camera exposure time of 100 ms, and an overall time-lapsed frame rate of 2 s^–1^ were selected. The lower iSCAT laser power and longer exposure time in the SP experiments were chosen to minimize photoreduction of the absorption tail of the blue excitable SP by the longer (red)-wavelength iSCAT beam.

### Data Analysis

Data analysis was conducted in Python. Following the camera dark count correction, temporal fluctuations in laser intensity were removed via division of each frame by frame modal pixel value. Spatial normalization was then performed using a median of 100 frames corresponding to the time before AuNPs are detectable. We performed no further temporal binning of our data. AuNPs were finally tracked using the Python module TrackPy (github.com/soft-matter/trackpy).^63^ The contrast of individual particles was determined as the maximum modulus intensity of each tracked object. Particle contrast trajectories were then subsequently fit to FW-model using scipy.optimize^64^ to produce the final processed data.

## Supporting Information Available

Real-time recording of individual AuNP formation; additional methods and image analysis steps; further details of the experimental apparatus; SEM images of AuNPs; instrument calibration; and fitting parameter distributions (PDF)
Real-time recording (MP4)
