# Supplementary material for: Real-Time Monitoring and Control of Nanoparticle Formation
Source: J Am Chem Soc. 2023 Jul 17;145(29):15809–15. doi: 10.1021/jacs.3c02484 (PMC10375529; doi:10.1021/jacs.3c02484)
Supplement: Supplementary file 1 — ja3c02484_si_001.pdf [file ja3c02484_si_001.pdf]

Real-time monitoring and control of nanoparticle formation

Yujie Guo[1], Vivien Walter[2], Steven Vanuytsel[1], Christopher Parperis[1], Jason T. Sengel[1], Eve E. Weatherill[1], and Mark I. Wallace[1]

[1]Department of Chemistry, King's College London, Britannia House, 7 Trinity Street, London, SE1 1DB, UK
[2]Department of Engineering, King's College London, London, WC2R 2LS, UK

Supplementary movies

Movie S1

Raw data of AuNP growth monitored by iSCAT microscopy. As NPs grow, they are first detected with contrast more negative than the overall background (dark spots) before becoming positive (bright spots). We also observed that for some particles, as the particle becomes very large, the AuNP detaches from the surface. 0.4 mM $HAuCl_4$ and 1 mM citrate were used (scale bar 2 µm). A laser power density of 0.003 mW µm^{-2} at 637 nm, exposure time 220 µs and overall time-lapsed frame rate 1 s^{-1} were chosen. This movie corresponds to the data in Fig. 2.

Supplementary methods

iSCAT contrast variation with nanoparticle diameter

iSCAT contrast is described in terms of the particle scattering cross-section (σ_{scat}) in Eq. 2 in the main body of the manuscript, which further relates to the particle polarizibility (α). Assuming a spherical particle, α and σ_{scat} can be further broken down as:

$$\alpha = \frac{\pi D^3}{2}\left(\frac{n_{\mathrm{p}}^2 - n_{\mathrm{m}}^2}{n_{\mathrm{p}}^2 + 2n_{\mathrm{m}}^2}\right) \tag{S1}$$

$$\sigma_{\mathrm{scat}} = \frac{8\pi^3}{3}\left(\frac{n_{\mathrm{m}}}{\lambda}\right)^4 \alpha^2 = P_1 D^6 \tag{S2}$$

where D is particle diameter, n_{p} and n_{m} are the refractive index of the particle and the surrounding medium, respectively, λ is the wavelength of illumination source [(i)]. To simplify the expression of σ_{scat}, it can be expressed as the product of parameter 1 (P_1) and diameter raised to the power of 6 (D^6).

[(i)]Dastjerdi, H. M.; Dahmardeh, M.; Gemeinhardt, A.; Mahmoodabadi, R. G.; Köstler, H.; Sandoghdar, V. Optimized Analysis for Sensitive Detection and Analysis of Single Proteins via Interferometric Scattering Microscopy. *J. Phys. D: Appl. Phys.* **2021**, 55 (5), 054002.

The diameter of the NP is described by FW-model in Eq. 1 in the main body of the manuscript. Thus, the relation between contrast and particle diameter can be determined and linked to the initial experimental conditions. Combining Eq. 1, Eq. 2, Eq. S1 and Eq. S2, yields a relation between the reaction kinetics and the contrast detected by iSCAT:

$$c = \beta^2 P_1 \left(D_\mathrm{f}^3 \left(1 - \frac{k_1 + k_2[\mathrm{A}]_0}{k_2[\mathrm{A}]_0 + k_1 e^{(k_1 + k_2[\mathrm{A}]_0)t}} \right) \right)^2 - 2\beta\sqrt{P_1} D_\mathrm{f}^3 \left(1 - \frac{k_1 + k_2[\mathrm{A}]_0}{k_2[\mathrm{A}]_0 + k_1 e^{(k_1 + k_2[\mathrm{A}]_0)t}} \right) \sin\varphi \tag{S3}$$

Data analysis

Image analysis consisted of the following steps:

1. Image stacks were first cropped to remove non-laser illumination area.
2. Dark counts subtraction was conducted by subtracting each frame by a frame recorded under the same conditions without laser illumination.
3. Laser intensity fluctuations were suppressed by dividing each frame by its modal pixel value.
4. To convert image intensities to contrast values, images were then background normalized via division with an image corresponding to the median-average of 100 frames corresponding to the image area prior to NP growth.
5. Particles were located using the Python module TrackPy [(ii)]. Particles were selected based on the following filtering conditions: the diameter of the circle used to detect and analyze spots was set to 21; a minimum separation between particles of 2× this diameter was specified; the minimum integrated brightness was set to 0.01 and a threshold with the value of 0.001 was applied. TrackPy returns information includes the position and the size of tracked objects. A region of interest (ROI) was defined by using the particle position as an origin and size as diameter. Then within the ROI the pixel value with the maximum absolute value was defined as contrast.
6. The contrast evolution of each tracked particle was fitted to Eq. S3 using scipy.optimize with fixed parameters ($n_\mathrm{p} = 0.18$, $n_\mathrm{m} = 1.33$, $\beta = 9.07 \times 10^7$ m^{-1}) as determined from calibration with AuNP of known diameter (Fig. S3).
7. Fitted NP tracks were then filtered to exclude outliers corresponding to situations where two NPs were too close to be distinguished, or where growth period was too short to provide enough data points for fitting. k_1, k_2 and D_f were restricted to positive values. φ was restricted between 0 to 2.3.

[(ii)] github.com/soft-matter/trackpy

Supplementary figures

Figure S1

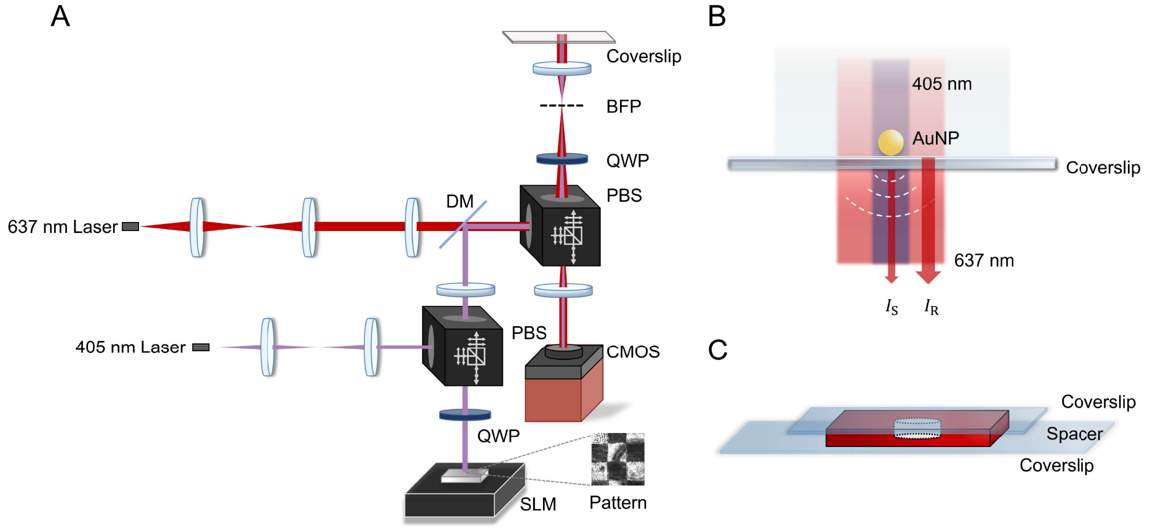

Figure S1: **Details of apparatus.** **(A)** iSCAT setup with a checkerboard pattern applied to the spatial light modulator (SLM). DM, dichroic mirror; PBS, polarizing beam splitter; QWP, quarter-wave plate; BFP, back focal plane; CMOS, complementary metal oxide semiconductor sensor. **(B)** Illustration of iSCAT detection at the sample under SLM illumination. **(C)** Construction of coverslip reaction chamber.

Figure S2

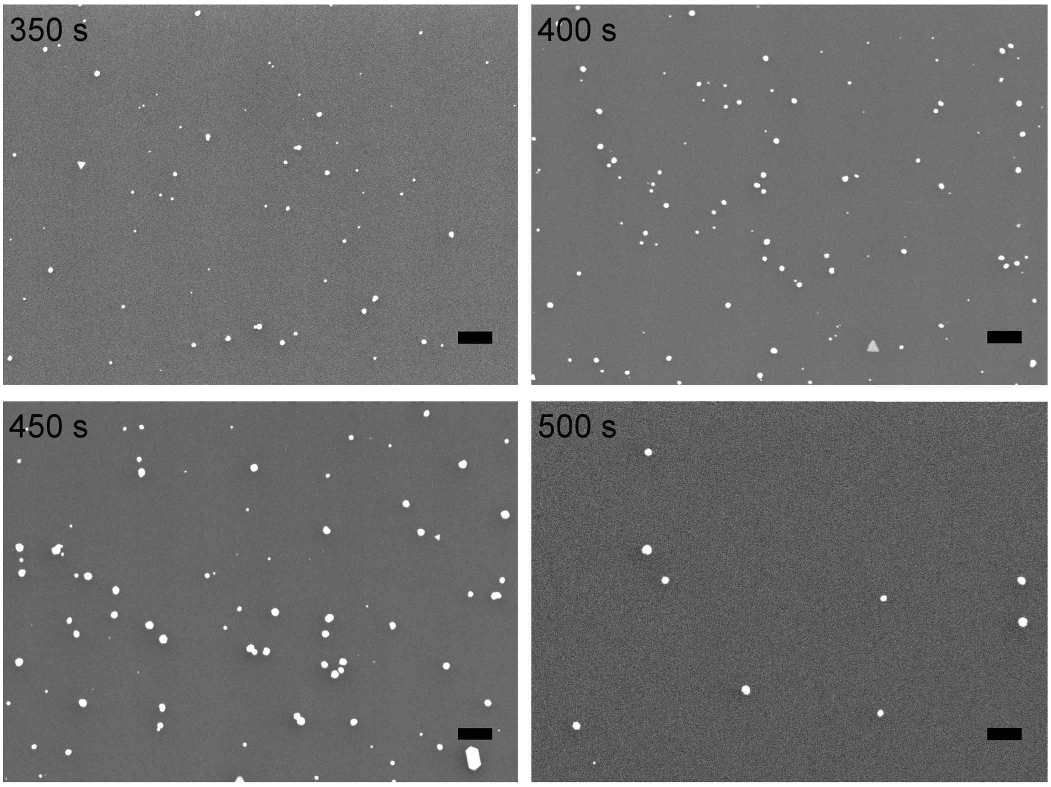

Figure S2: **SEM images of AuNPs produced via photocontrolled method at different time points.** Under the conditions of $HAuCl_4$ 0.6 mM, SP 0.3 mM and LED intensity 1.17 mW cm^{-2} (scale bar 400 nm).

Figure S3

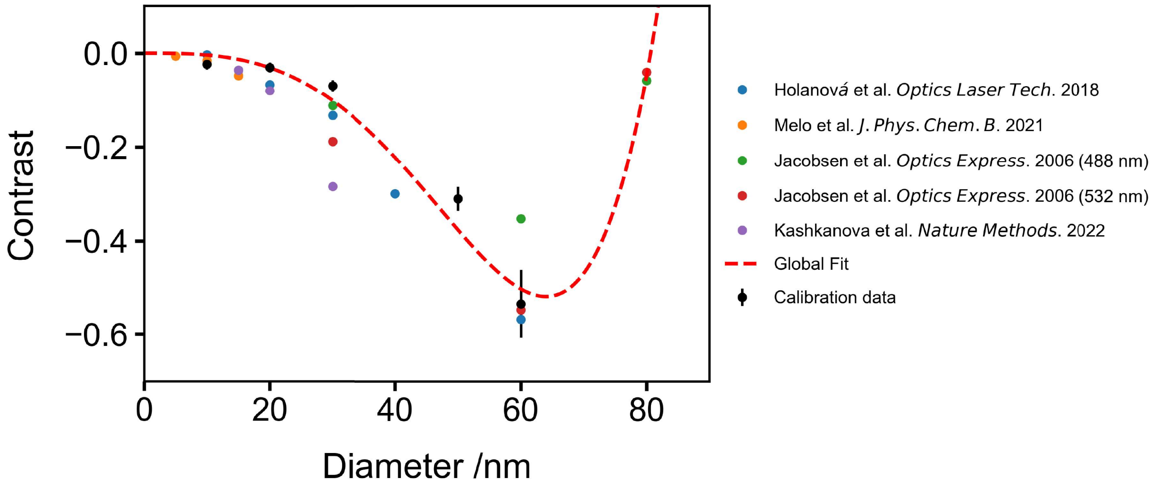

Figure S3: **Standard AuNPs calibration.** For instrument calibration, around 100 AuNPs of known size were tracked. The contrast distribution for each size was then fitted to a Gaussian function to determine the mean and the standard deviation. The evolution of mean contrast as function of particle size calibration curve is plotted alongside previously reported contrast values.[51,53,(iii,iv)] Error bars represent the standard deviations from our distribution of particle contrast. The calibration curve was then fitted to Eq. 2 using scipy.optimize with fixed refractive indices corresponding to our experiment ($n_{\mathrm{p}} = 0.18$ and $n_{\mathrm{m}} = 1.33$). Using this procedure β was determined as 9.07 $\times 10^{7}\,\mathrm{m}^{-1}$, similar to previously reported values.[51]

(iii) Melo, L.; Hui, A.; Kowal, M.; Boateng, E.; Poursorkh, Z.; Rocheron, E.; Wong, J.; Christy, A.; Grant, E. Size Distributions of Gold Nanoparticles in Solution Measured by Single-Particle Mass Photometry. *J. Phys. Chem. B* **2021**, 125 (45), 12466–12475.

(iv) Jacobsen, V.; Stoller, P.; Brunner, C.; Vogel, V.; Sandoghdar, V. Interferometric Optical Detection and Tracking of Very Small Gold Nanoparticles at a Water-Glass Interface. *Opt. Express* **2006**, 14 (1), 405.

Figure S4

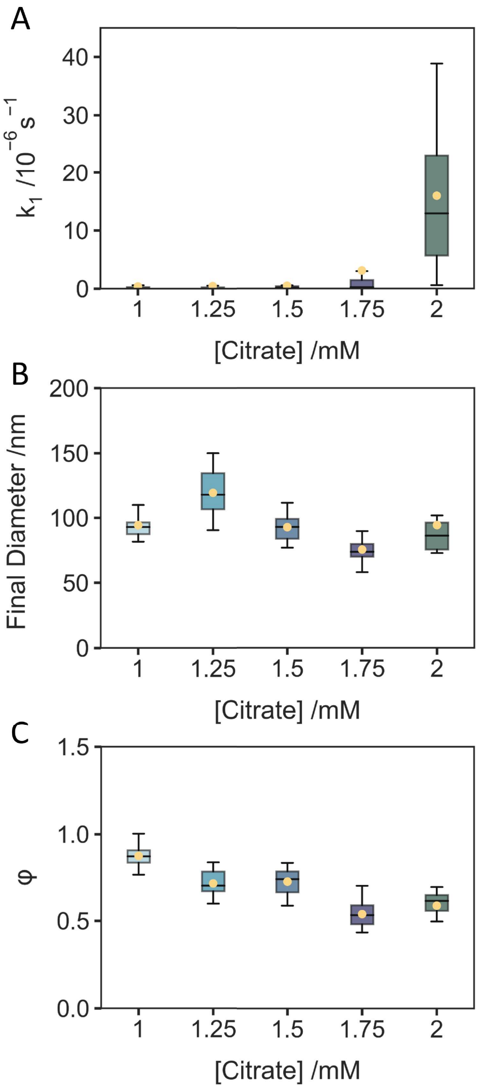

Figure S4: **Citrate synthesis of AuNPs: additional fitting parameters.** Citrate was used as the reductant with concentration varied from 1 to 2 mM, $HAuCl_4$ concentration was fixed at 0.4 mM. **(A)** Effect of citrate concentration on nucleation rate constant (k_1). **(B)** Effect of citrate concentration on final diameter (D_f) of individual AuNPs. **(C)** Effect of citrate concentration on the Gouy phase shift between reference and scattered field (φ).

Figure S5

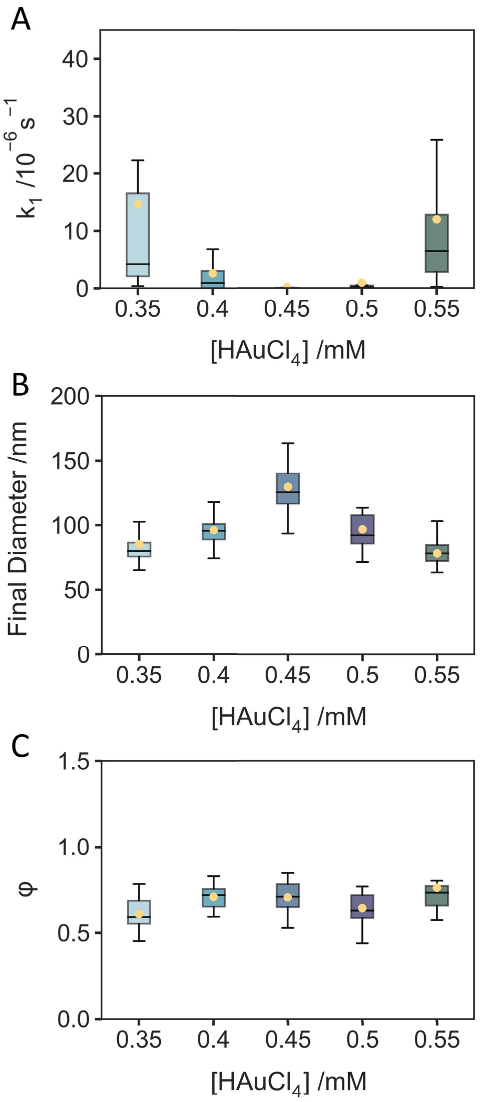

Figure S5: **Citrate synthesis of AuNPs: additional fitting parameters.** Citrate was used as the reductant with the concentration fixed at 1.5 mM, $HAuCl_4$ concentration was varied from 0.35 to 0.55 mM. **(A)** Effect of $HAuCl_4$ concentration on nucleation rate constant (k_1). **(B)** Effect of $HAuCl_4$ concentration on final diameter (D_f) of individual AuNPs. **(C)** Effect of $HAuCl_4$ concentration on the Gouy phase shift between reference and scattered field (φ).

Figure S6

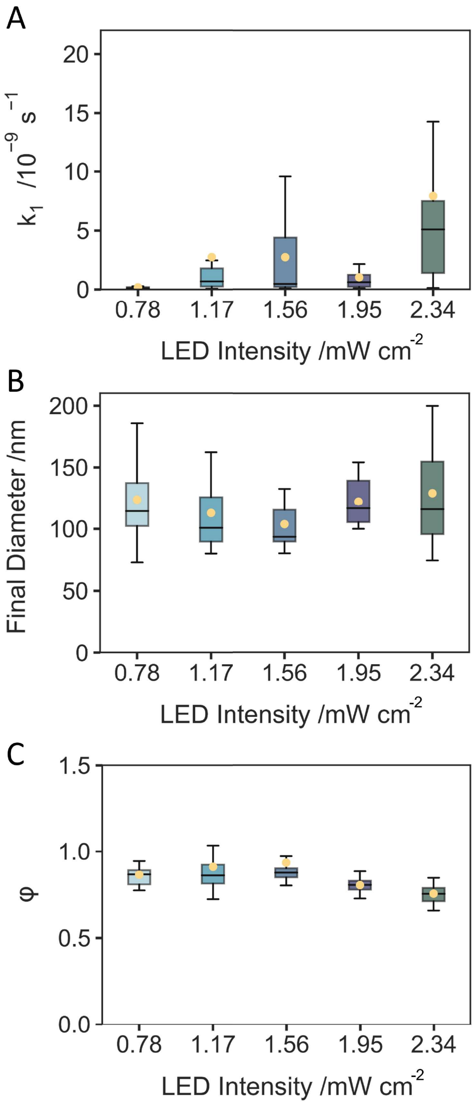

Figure S6: **AuNP formation using SP photocontrol: additional fitting parameters.** The concentration of SP and $HAuCl_4$ were fixed at 0.35 and 0.6 mM, respectively, and the photoreduction illumination intensity varied from 0.78 to 2.34 mW cm^{-2}. **(A)** Effect of LED intensity on nucleation rate constant (k_1). **(B)** Effect of LED intensity on final diameter (D_f) of individual AuNPs. **(C)** Effect of LED intensity on the Gouy phase shift between reference and scattered field (φ).

Figure S7

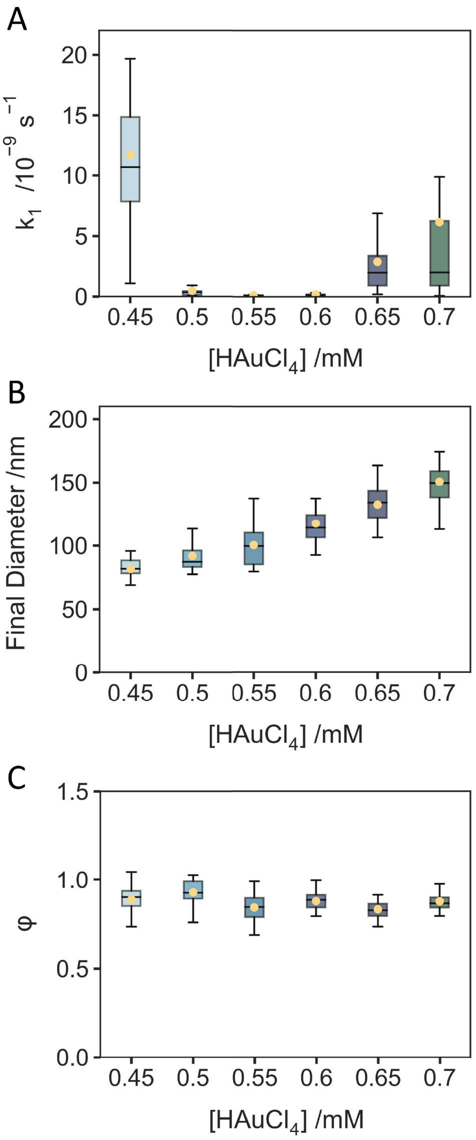

Figure S7: **AuNP formation using SP photocontrol: additional fitting parameters.** SP concentration was fixed at 0.35 mM and the LED intensity was maintained at 1.17 mW cm^{-2}, the $HAuCl_4$ concentration was varied from 0.45 to 0.7 mM. **(A)** Effect of $HAuCl_4$ concentration on nucleation rate constant (k_1). **(B)** Effect of $HAuCl_4$ concentration on final diameter (D_f) of individual AuNPs. **(C)** Effect of $HAuCl_4$ concentration on the Gouy phase shift between reference and scattered field (φ).

Figure S8

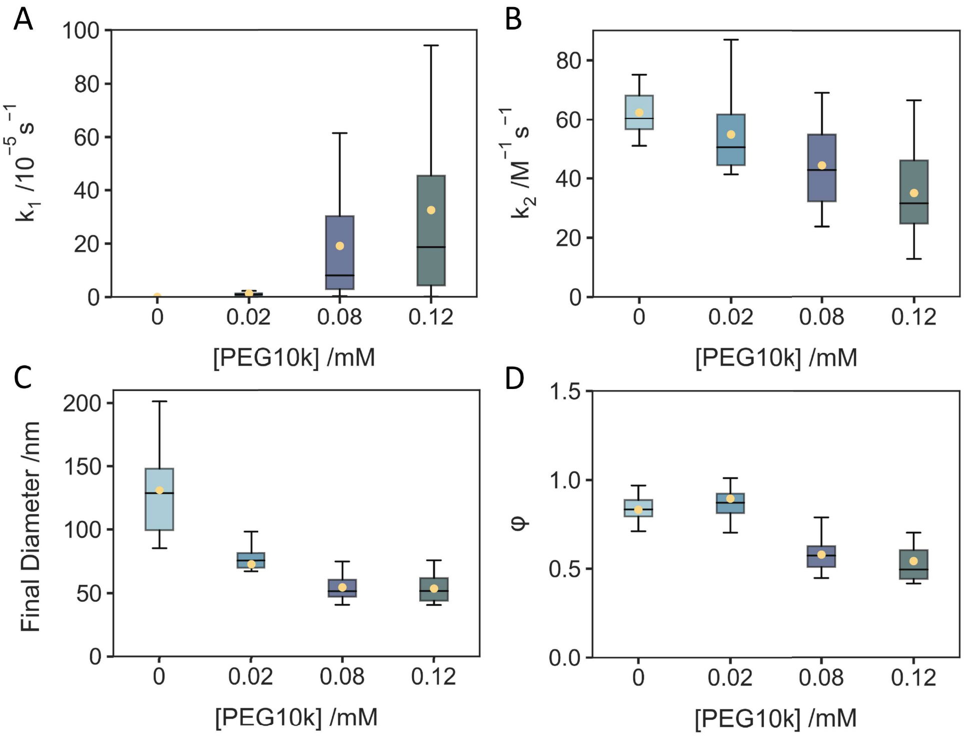

Figure S8: **The impact of adding PEG10k as a capping agent.** The role of PEG10k as a capping agent was investigated, varying the PEG10k concentration under otherwise identical photoreductive growth conditions using SP as per the main text (0.35 mM SP, 0.6 mM $HAuCl_4$ and LED intensity of 1.95 mW cm^{-2}). **(A)** Effect of PEG10k concentration on nucleation rate constant (k_1). As the concentration of PEG10k increases, the nucleation process of the AuNP formation gradually accelerates. **(B)** Effect of PEG10k concentration on autocatalytic growth rate constant (k_2). As the concentration of PEG10k increases, the autocatalytic growth rate gradually decreases. **(C)** Effect of PEG10k concentration on the final particle diameter (D_f) of individual AuNPs. By coating the NP surface, the capping agent enables inhibition of NP over-growth, leading to a decrease in particle size. **(D)** Effect of PEG10k concentration on the Gouy phase shift between reference and scattered field (φ). As the concentration of PEG10k increases, φ gradually decreases.
